# WholeCellSimDB: a hybrid relational/HDF database for whole-cell model predictions

**DOI:** 10.1093/database/bau095

**Published:** 2014-09-16

**Authors:** Jonathan R. Karr, Nolan C. Phillips, Markus W. Covert

**Affiliations:** ^1^Graduate Program in Biophysics, Stanford University, Stanford, CA 94305, USA, ^2^Computer Science and Information Technology, University of Prince Edward Island, Charlottetown, PE C1A 4P3, Canada and ^3^Department of Bioengineering, Stanford University, Stanford, CA 94305, USA

## Abstract

Mechanistic ‘whole-cell’ models are needed to develop a complete understanding of cell physiology. However, extracting biological insights from whole-cell models requires running and analyzing large numbers of simulations. We developed WholeCellSimDB, a database for organizing whole-cell simulations. WholeCellSimDB was designed to enable researchers to search simulation metadata to identify simulations for further analysis, and quickly slice and aggregate simulation results data. In addition, WholeCellSimDB enables users to share simulations with the broader research community. The database uses a hybrid relational/hierarchical data format architecture to efficiently store and retrieve both simulation setup metadata and results data. WholeCellSimDB provides a graphical Web-based interface to search, browse, plot and export simulations; a JavaScript Object Notation (JSON) Web service to retrieve data for Web-based visualizations; a command-line interface to deposit simulations; and a Python API to retrieve data for advanced analysis. Overall, we believe WholeCellSimDB will help researchers use whole-cell models to advance basic biological science and bioengineering.

**Database URL:** http://www.wholecellsimdb.org

**Source code repository URL:** http://github.com/CovertLab/WholeCellSimDB

## Introduction

Mechanistic ‘whole-cell’ models are essential for developing a complete understanding of cell physiology and enabling rational bioengineering and personalized medicine (1). Whole-cell models are needed to guide and interpret experiments, unify our collective knowledge of cell physiology and form the basis of computer-aided biological design and medical decision-making software. However, constructing whole-cell models is a major engineering challenge.

Recently Karr *et al.* demonstrated a novel integrative modeling methodology, which promises to enable researchers to build comprehensive models of individual cells (2). Karr *et al.* used this integrative modeling approach to construct the first whole-cell model of the gram-positive bacterium *Mycoplasma genitalium*. The model is composed of 28 submodels of every major intracellular process in *M. genitalium*. The model predicts the dynamics of every molecular species across the life cycle of a single *M. genitalium* cell. They have used the model to generate novel hypothesis about *Mycoplasma* cell cycle and energy regulation (2) and learn unknown kinetic parameters (3).

Gaining new biological insights from whole-cell simulations requires analyzing terabytes of predicted phenotypic data. This in turn requires systems that allow researchers to quickly retrieve model predictions for visualization and analysis.

Databases are routinely used to manage and share biological data. For example, structural biologists use The Protein Data Bank to store protein structures (4), geneticists use GenBank to share genomic sequences (5) and genomics researchers use the Gene Expression Omnibus to organize gene expression profiles (6) among many experimental databases routinely used in biology. Databases including KEGG (7), NCBI Genome (8) and UniProt (9) are commonly used to manage genome annotations. Pathway/genome databases including BiGG (10), BioCyc (11) and WholeCellKB (12) are frequently used to construct computational models. Model databases including BioModels (13) and the CellML Model Repository (14) are regularly used to share computational models.

Despite their popularity and ease of use, databases have not been widely adopted for organizing simulation results data, in part because relational databases are not well suited for matrix-based data. Only a few researchers have used databases to store molecular dynamics (15–18), cosmological (19, 20) and systems biology (21) simulations.

We developed WholeCellSimDB, a hybrid relational/hierarchical data format (HDF) database, to help researchers organize and retrieve whole-cell simulation setup data and results data for further analysis. WholeCellSimDB was primarily designed to enable individual research groups to privately organize their own simulations. However, WholeCellSimDB also enables researchers to share simulations with the broader scientific community through a simple Web-based interface.

WholeCellSimDB uses a relational database to store all of the simulation setup metadata required to reproduce a simulation and HDF5 to store every model prediction. This enables researchers to quickly search, sort and compare simulation setup metadata as well as quickly retrieve large amounts of simulation results data. WholeCellSimDB is consistent with the minimum information about a simulation experiment guidelines (22).

WholeCellSimDB provides researchers four user interfaces to deposit, explore and retrieve metadata and results data: a Web-based graphical interface, a Web service, a command-line interface and a Python API. The Web-based graphical interface enables researchers to search, browse and compare simulations by metadata, drill down to individual phenotype predictions and export results data in HDF5 format and metadata in SED-ML format (23). The graphical interface also provides a simple data viewer, which enables researchers to plot predicted phenotype dynamics. The JavaScript Object Notation (JSON) Web service interface can be used to enable more sophisticated visualizations and analyses such as WholeCellViz (24). The command-line interface enables researchers to deposit simulations into WholeCellSimDB. Finally, the Python API provides complete access to WholeCellSimDB, including the capability to deposit, retrieve and export simulations. Here, we describe WholeCellSimDB’s implementation, features and usage.

## Implementation

### Software architecture

WholeCellSimDB is composed of a database server and a Web-based graphical user interface. The database server stores whole-cell results data, as well as all of the metadata needed to reproduce each simulation. The user interface allows researchers to browse, search and plot the results data and metadata.

The results data consist of every predicted phenotype at every simulation time point, including the predicted mass; metabolite, RNA and protein copy numbers; and DNA-binding proteins locations. The metadata consists of the location of the model’s source code repository, the name of the revision, which was simulated, the name of investigator who simulated the model, the time when the simulation was executed and the chosen values of every option and parameter.

To efficiently search metadata as well as efficiently retrieve results data, we implemented WholeCellSimDB using a hybrid relational/HDF5 architecture. We used a relational database to store metadata. This enables WholeCellSimDB to quickly and easily search and sort metadata using relational database queries. We used HDF5 (25) to store the much larger volume of results data because HDF5 supports hierarchical data, compression and chunking and, therefore, can compactly store as well as quickly retrieve results data. In addition, HDF5 has bindings for all of the most commonly used scientific programming languages. In particular, we chose HDF5 rather than NetCDF because HDF5 better supports larger data sets and hierarchical data and provides compression.

The database server and user interface communicated using JSON through an Apache Web server (http://httpd.apache.org) and mod_wsgi (http://code.google.com/p/mod wsgi). The user interface requested results data and metadata using JSON, and the database server used JSON to provide the user interface simulation results and metadata.

Overall, WholeCellSimDB’s hybrid relational/HDF5 architecture is similar to the hybrid XML/HDF5 design of SDCubes (26). However, WholeCellSimDB uses a relational database to store metadata, whereas SDCubes uses a collection of XML documents. This allows WholeCellSimDB to take advantage of well established relational database frameworks to provide researchers searching and sorting capabilities and a Web-based graphical interface not present in SDCubes. In particular, the Web-based graphical interface allows researchers to use WholeCellSimDB to share results data with the broader scientific community. WholeCellSimDB’s architecture is also similar to the hybrid architecture of SEEK (21); however, WholeCellSimDB stores all results in HDF5 format rather than Excel or XML to enable researchers to efficiently store and retrieve large data sets. To compare results from multiple simulations, WholeCellDB also stores results data using a specific HDF5 schema. In contrast, because SEEK does not require any specific schema, SEEK is unable to facilitate comparisons among multiple simulations.

### Data model

WholeCellSimDB organizes simulations into *in silico* organisms, which are subdivided into versions and further subdivided into batches. *In silico* organisms are computational models of biological organisms. Typically, each *in silico* organism corresponds to a distinct source code repository and set of default parameter values, and each version corresponds to a specific revision of the repository as the researchers iteratively develop their model. Simulation batches are groups of simulations, which correspond to populations of genetically identical *in silico* cells. The simulations within each batch share the same model code and parameter values, and differ only in their random number generator seed values. Consequently, for simplicity, WholeCellSimDB stores option and parameter values and all other metadata at the simulation batch level. Although this design choice causes WholeCellSimDB to redundantly store unchanged option and parameter values, because the metadata is much smaller than the results data, the additional storage requirements are negligible.

### Database server

The database server was implemented in Python using the Django framework (http://www.djangoproject.com), a MySQL relational database (http://www.mysql.com) and h5py (http://www.h5py.org). Full-text search was implemented using Haystack (http://haystacksearch.org), Xapian (http://xapian.org) and Google Custom Search (http://www.google.com/cse).

### User interface

The WholeCellSimDB Web-based user interface was implemented in HTML5 and JavaScript using jQuery (http://jquery.com). User interface widgets were implemented using jQWidgets (http://www.jqwidgets.com) and jqTree (http://mbraak.github.io/jqTree). Plotting was implementing using Flot (http://www.flotcharts.org).

### Source code

The WholeCellSimDB source code is available open source under the MIT license at http://github.com/CovertLab/WholeCellSimDB.

## Usage

**Figure 1.**
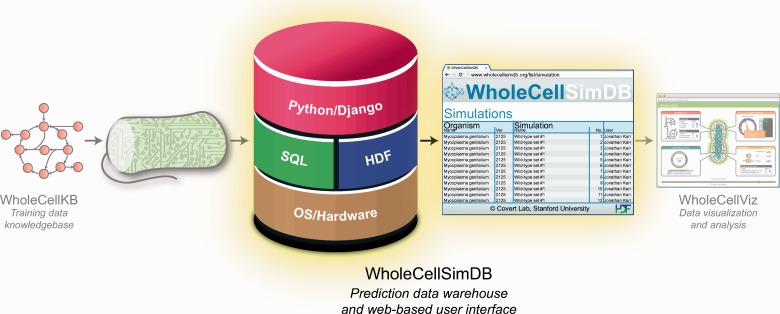
WholeCellSimDB (center) stores whole-cell model simulations for visualization and analysis. WholeCellSimDB is composed of a hybrid relational/HDF database server and a Web-based user interface. Researchers contribute whole-cell model simulations to WholeCellSimDB via a command line interface (left), and retrieve results data for further analysis using either the JSON Web service or Python API (right).

WholeCellSimDB was designed to help researchers analyze whole-cell simulation data by enabling them to browse metadata and quickly retrieve subsets of results data. Researchers deposit whole-cell simulations into WholeCellSimDB via a command-line interface, and can use the WholeCellSimDB Web service to power analysis tools such as WholeCellViz (24) (Figure 1).

### Data deposition

After running and saving simulations, researchers can use the command-line interface to deposit simulations into WholeCellSimDB. The command-line interface requires an HDF5 file, which describes the model predictions. The HDF5 file must follow the schema described at http://www.wholecellsimdb.org/help. WholeCellSimDB provides a utility to convert simulations stored using the MATLAB MAT-File binary format described in Karr *et al.* 2012 (2) to the required HDF5 format. In addition, the command-line interface requires either an HDF5 or SED-ML (23) file, which describes the chosen changes to the default option and parameter values. The command-line interface copies the submitted HDF5 file to a separate directory, and records all of the changes to the default option and parameter values into the relational database. All deposited simulations are publicly accessible.

Owing to the large size of whole-cell simulation data, deposition to WholeCellSimDB.org is currently limited to the authors’ research group. Researchers can use the WholeCellSimDB software to store their own simulations by installing the software on their own machines. Furthermore, researchers can use Apache basic authentication restrict access to their simulations to specific users and/or groups.

After a simulation is deposited, it is permanently stored and can never be deleted or modified. The HDF5 files and relational database are both periodically backed up to secondary server.

### Web-based graphical interface

Next, researchers can use the Web-based interface to explore metadata and identify simulations for further analysis. The Web interface provides researchers several ways to explore stored simulations. First, the Web interface provides pages listing all of the stored *in silico* organisms and simulations. These pages provide hyperlinks to drill down to individual simulation pages listing their option and parameter values, modeled cellular processes and predicted phenotypes.

**Figure 2.**
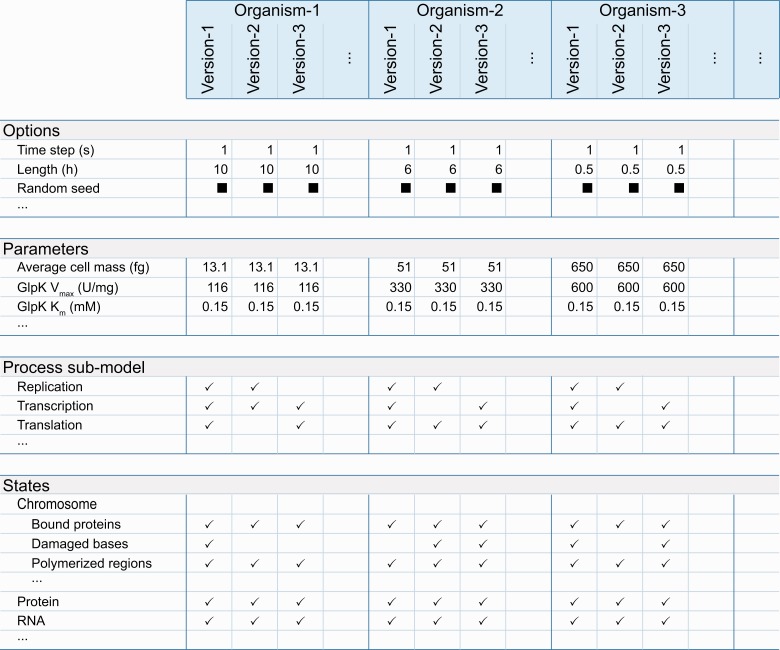
The WholeCellSimDB Web interface enables researchers to browse metadata to find simulations for further analysis. WholeCellSimDB option, parameter, process submodel and state metadata summary views display all of the metadata (rows) for each simulation, grouped by *in silico* organism (columns). To highlight the differences among the simulations associated with each *in silico* organism, WholeCellSimDB only displays values that are identical across all simulations within an organism. Values that vary across simulations are indicated by black boxes. Blank entries indicate options, parameters, submodels and states, which are not used by any simulation within an organism. The row and column labels provide hyperlinks to more detailed views, which display the values of each individual option, parameter, state and process for each individual simulation. Interactive versions of the option, parameter, process submodel and state metadata summary views are available at http://www.wholecellsimdb.org.

The Web interface also provides tables, which compare simulation option and parameter values, modeled process submodels and categories of predicted phenotypes (e.g. metabolite concentrations, protein copy numbers) or states (Figure 2). These tables use one column to summarize the properties associated with each stored *in silico* organism. The tables display values that are identical across all associated simulations. Checkmarks indicate submodels and phenotypes predicted for each associated simulation; black boxes indicate properties, which vary across the associated simulations. These tables provide hyperlinks to drill down to individual options, parameters, process submodels and phenotypes. The individual option, parameter, and process submodel pages display each parameter, option and submodel’s value for every stored simulation. The predicted phenotypic category pages provide more detailed comparison tables, which summarize the specific phenotypes (e.g. individual metabolite concentrations, individual gene product copy numbers) predicted for each *in silico* organism. These pages also provide researchers hyperlinks to further drill down to pages, which plot predicted phenotypes for specific subcellular compartments for specific simulation batches.

The Web interface provides three search tools: full-text search using either the WholeCellSimDB search engine or Google, and an advanced structured search tool. The advanced search tool provides researchers a Web form to find simulations with specific option and parameter values, modeled cellular process submodels and predicted phenotypes. The structured search tool was implemented using a Django Web form and the Django QuerySet API.

**Figure 3.**
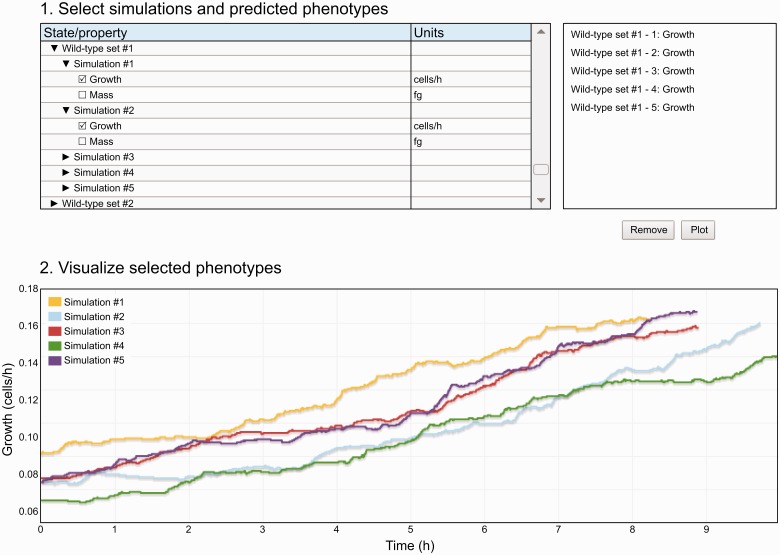
The WholeCellSimDB data viewer allows researchers to preview simulations stored in WholeCellSimDB. Researchers use the hierarchical simulation menu (top-right panel) to select simulations and predicted phenotypes. The top-right panel lists the selected simulations and phenotypes. The WholeCellSimDB viewer retrieves the selected simulations and phenotypes from the WholeCellSimDB server and creates an interactive plot (bottom panel). An interactive version of Figure 3 is available at http://www.wholecellsimdb.org.

In addition, the Web interface provides a data viewer (Figure 3), which enables researchers preview results data before deeper analysis. The data viewer allows researchers to select and plot small subsets of simulation data across one or more simulations. This functionality is similar to that provided by the Cooper *et al.* functional curation prototype (27). The data viewer is available on the WholeCellSimDB home page and on the individual simulation pages.

### Simulation data retrieval

Lastly, WholeCellSimDB provides three methods for researchers to export results data for further analysis. First, researchers can use the download utility or the download links provided in the Web interface. This allows researchers to download entire simulations and groups of simulations in HDF5 format, as well as slices of results data in HDF5 format. WholeCellSimDB provides entire simulations and batches only in HDF5 format because HDF5 is one of the few standard numerical data formats capable of compactly representing the large hierarchical data sets produced by whole-cell simulations. WholeCellSimDB does not provide entire simulations in NetCDF format because it does not support compression. WholeCellSimDB also does not provide entire simulations in text-based formats such as JSON or the Numerical Markup Language (NuML) because these formats are too inefficient for large data sets. To maximize the portability of the metadata, researchers can also download metadata in SED-ML format (23), the only standard simulation experiment language used in systems biology.

Second, WholeCellSimDB provides a Web service for retrieving slices of results data. To provide maximum compatibility with other software, WholeCellDB provides these data in several standard formats including HDF5, JSON, binary JSON (BSON), MessagePack and NuML (see http://www.wholecellsimdb.org/help for more information). This Web service can be used to power Web-based visualizations and analyses such as WholeCellViz (24).

Third, researchers can use the WholeCellSimDB Python API to retrieve simulation slices as NumPy arrays (28). The Python API is the fastest and flexible method and is best for complex analyses. The Web interface and Web service are publicly accessible. The Python API, however, is restricted to the authors’ own research group. Researchers can use the Python API to organize their own simulations by installing WholeCellSimDB on their own machines.

## Conclusions

We developed WholeCellSimDB to help researchers analyze whole-cell model simulation data by enabling researchers to easily find and retrieve results data. We primarily designed WholeCellSimDB to enable individual research groups to privately organize their own simulations. However, researchers can also use WholeCellSimDB to share simulations with the broader scientific community. WholeCellSimDB provides researchers several interfaces to explore stored simulations, including a Web-based graphical interface. WholeCellSimDB features a hybrid relational/HDF architecture, which enables researchers to both quickly search metadata as well as quickly retrieve large amounts of results data.

Going forward, we hope to expand WholeCellSimDB’s capabilities as a data analysis tool. In particular, we hope to extend WholeCellSimDB’s search capability to predicted phenotype values. This would enable researchers to analyze whole-cell simulations in both forward and reverse directions. Researchers would be able to analyze the impact of parameters on the predicted phenotypes as well as identify parameters that produce specific phenotypes. In particular, we hope to develop a simple language for querying the predicted phenotypic values, optimize WholeCellSimDB’s disk usage and retrieval performance, and integrate WholeCellSimDB with graphical analysis tools such as WholeCellViz to enable more researchers to interact with whole-cell simulations. In addition, we plan to integrate WholeCellSimDB with WholeCellKB by (i) providing hyperlinks inside WholeCellSimDB from model parameters and predictions to their related experimental data in WholeCellKB, and (ii) adding lists of the simulated values corresponding to each experimental measurement to WholeCellKB.

Currently, WholeCellSimDB contains simulations of a whole-cell model of *M. genitalium* as well as simulations of three *Escherichia coli* models: an ordinary differential equation model of central carbon metabolism (29), a regulatory flux-balance analysis model of metabolism (30) and an integrated metabolic model (31). Going forward, we plan to deposit simulations of additional whole-cell models into WholeCellSimDB. Researchers can use WholeCellSimDB to organize their own simulations by running the software on their own machines, either by installing WholeCellSimDB or by running our whole-cell virtual machine available at http://simtk.org/home/wholecell.

In summary, WholeCellSimDB is a powerful tool for helping researchers analyze whole-cell simulations. We anticipate that WholeCellSimDB will help researchers realize the full potential of whole-cell models for biological discovery, bioengineering and medicine. More broadly, WholeCellSimDB serves as a valuable example of how to systematically organize and communicate results data, and how relational databases and HDF can be combined to manage scientific data.
